# Supplementary material for: The molecular basis of FimT-mediated DNA uptake during bacterial natural transformation
Source: Nat Commun. 2022 Mar 4;13:1065. doi: 10.1038/s41467-022-28690-1 (PMC8897410; doi:10.1038/s41467-022-28690-1)
Supplement: Supplementary file 1 — Supplementary Information [file 41467_2022_28690_MOESM1_ESM.pdf]

## Supplementary Information

# The Molecular Basis of FimT-mediated DNA uptake during Bacterial Natural Transformation


Sebastian A.G. Braus[1], Francesca L. Short[2,3], Stefanie Holz[1,#], Matthew J.M. Stedman[1,#], Alvar D. Gossert[1,4], Manuela K. Hospenthal[1,5,*]

[1] Institute of Molecular Biology and Biophysics, ETH Zürich, Otto-Stern-Weg 5, 8093 Zürich, Switzerland

[2] Department of Microbiology, Monash University, 19 Innovation Walk, Clayton, 3800, Victoria, Australia

[#] These authors contributed equally

* Correspondence: manuela.hospenthal@mol.biol.ethz.ch

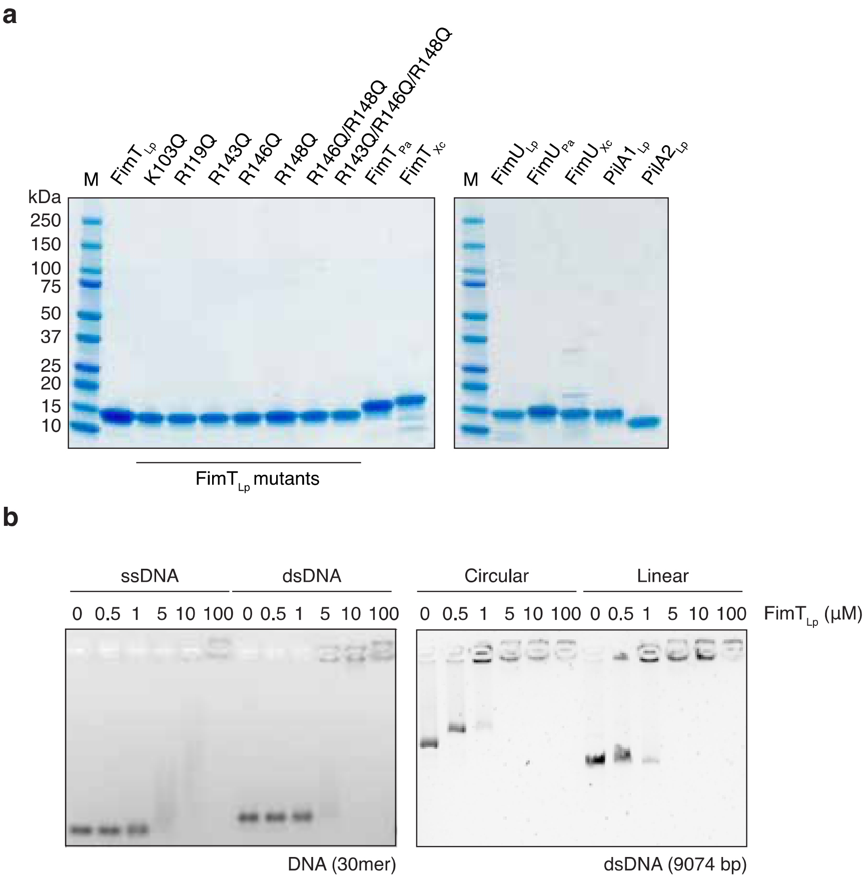


**Supplementary Figure 1: Purified proteins utilised in this study and *in vitro* DNA binding of $FimT_{Lp}$**

**a**, All purified N-terminally truncated pilins (construct boundaries can be found in Supplementary Table 3), utilised in this study, resolved by SDS-PAGE. M, marker; Lp, *L. pneumophila*; Pa, *P. aeruginosa*; Xc, *X. campestris*. **b**, EMSAs showing *in vitro* DNA binding of $FimT_{Lp}$ to ssDNA vs dsDNA (left) and linear vs circular DNA (right). DNA probes were incubated with increasing concentrations of $FimT_{Lp}$ and resolved by agarose gel electrophoresis. This experiment was independently performed three times with reproducible results.

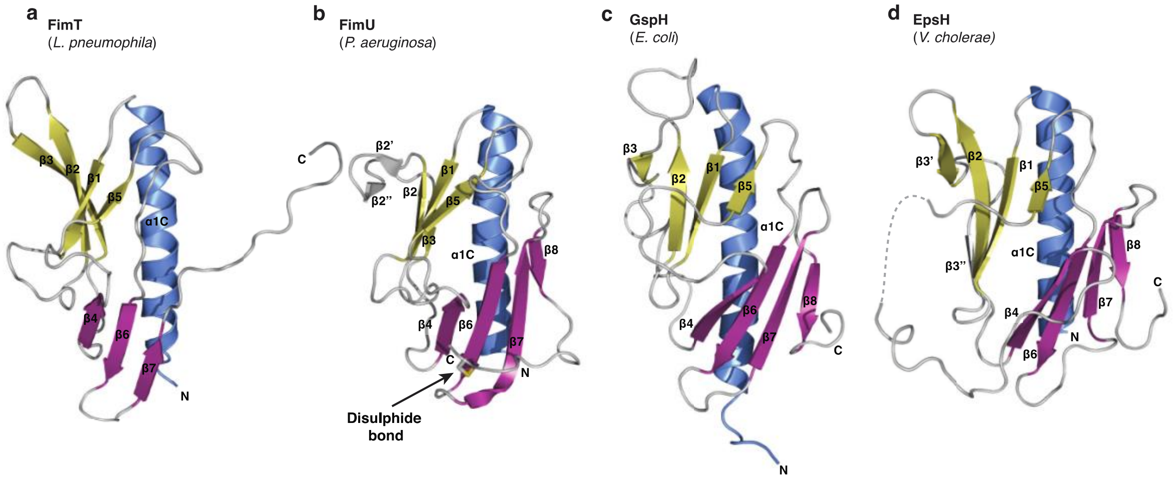


**Supplementary Figure 2: Structures of GspH/FimT family members**

**a**, The structure of FimT from *L. pneumophila* (state 18, this study); **b**, FimU from *P. aeruginosa* (PDB ID: 4IPV); **c**, GspH from *E. coli* (state 1, PDB ID: 2KNQ); and **d**, EpsH from *V. cholerae* (PDB ID: 2QV8). The $FimT_{Lp}$ and $GspH_{Ec}$ structures were determined using NMR spectroscopy, while those of $FimU_{Pa}$ and $EpsH_{Vc}$ are crystal structures. The disulphide bond of FimU is shown in stick representation (sulphur atoms in yellow), indicated by an arrow. The previously named β3 and β4 strands of the EpsH structure[1] have been labelled as β3' and β3'' for consistency of strand nomenclature across all depicted structures. All structures are shown in ribbon representation with their N-and C-termini indicated and secondary structural elements are coloured and labelled as in Fig. 2a.

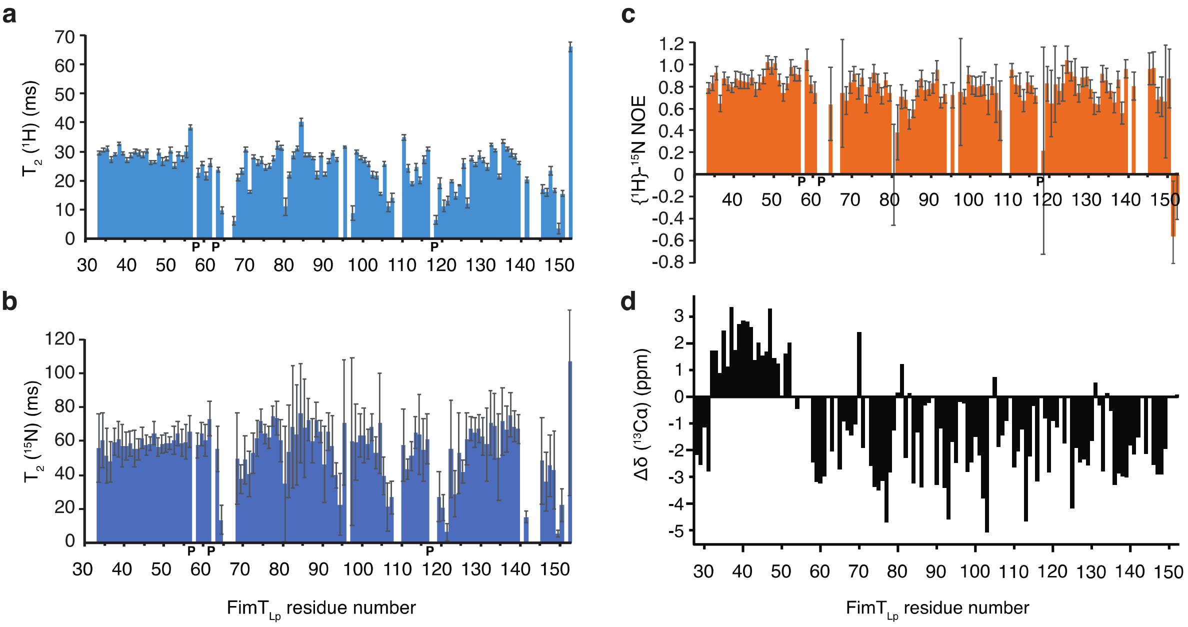


**Supplementary Figure 3: Relaxation data for FimT$_{Lp}$ indicate dynamics of the C-terminal residues 140–150 on the millisecond timescale**

**a**, **b**, Backbone amide $T_2$ transverse relaxation data of FimT$_{Lp}$ for $^1$H (**a**) and $^{15}$N (**b**) nuclei, where amide groups of the loops and the C-terminus show significantly decreased $T_2$ values compared to the folded part of the domain. The low $T_2$ values for the C-terminal tail (signals of amides of residues 140 and 142–144 were too weak to be analysed), indicate dynamics of the C-terminal residues (140–150) on the microsecond to millisecond timescale. Only the final residue exhibits a long $T_2$ value, which is typical for flexibly disordered termini. Proline residues are indicated with a bold letter P. Error bars represent the fitting errors of the respective exponential decay curves. **c**, Heteronuclear {$^1$H}-$^{15}$N NOE data also show that only the final two residues (151 and 152) exhibit fast dynamics on the nanosecond timescale. Error bars reflect the error from the signal-to-noise ratio of the individual signals used for the analysis. **d**, Cα chemical shift deviation from random coil values (Δδ ($^{13}$Cα)) indicate predominantly β-strand secondary structure for the C-terminal residues. Significant (> 0.5 ppm) positive and negative deviations of $^{13}$Cα chemical shifts from random coil values indicate α-helical and β-strand conformations of the backbone, respectively. $^{13}$Cα chemical shifts are shown without smoothing, representing the raw data after calibration of the $^{13}$C chemical shift to 2,2-dimethyl-2-silapentane-5-sulfonate (DSS).

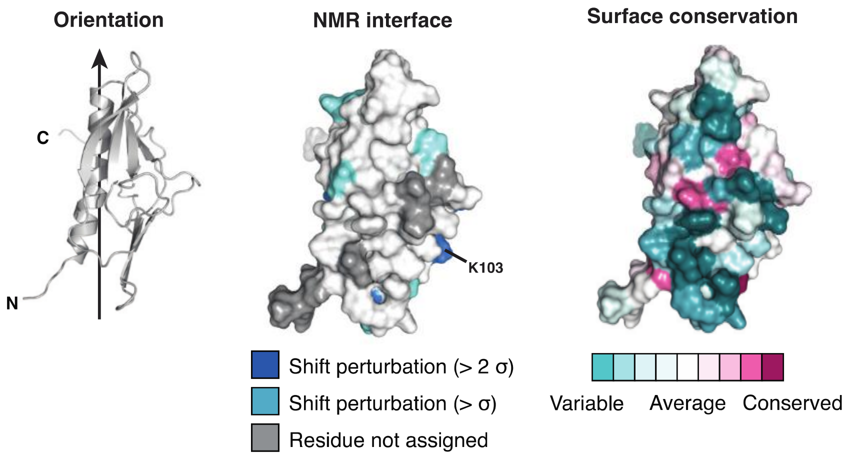


**Supplementary Figure 4: NMR binding studies of $FimT_{Lp}$ to DNA**

Left, $FimT_{Lp}$ is shown in ribbon representation rotated a further 120° with respect to the orientations displayed in Fig. 3c. Middle, residues experiencing chemical shift perturbations due to DNA binding are mapped onto the surface of $FimT_{Lp}$. Right, surface residues of $FimT_{Lp}$ are coloured according to conservation. This image was generated using the ConSurf server[2].

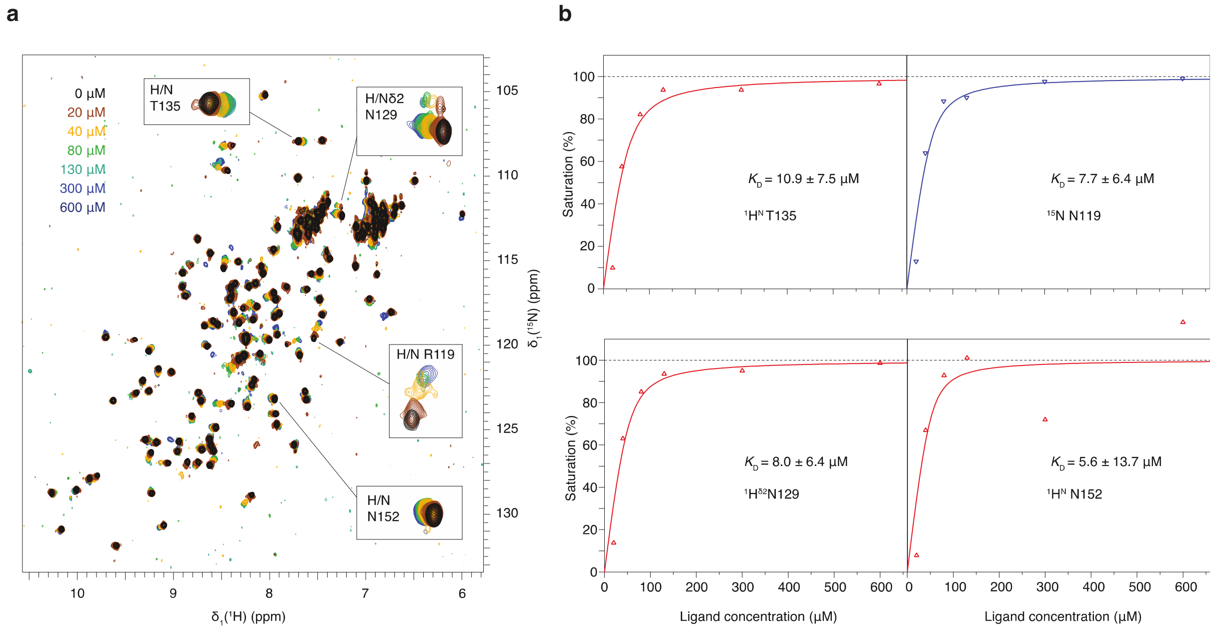


**Supplementary Figure 5: Affinity determination of FimT to 12 bp dsDNA by NMR**

**a**, DNA binding studies of $FimT_{Lp}$ performed by NMR spectroscopy. Increasing concentrations of 12 bp dsDNA (see colour code on top left in overlaid spectra) were added to 40 µM of $^{15}$N-labelled $FimT_{LP}$ and the CSPs of four peaks were plotted against the ligand (12 bp DNA) concentration. **b**, For the four signals indicated in the overlaid spectra, the binding curves are shown on the right-hand side, for $^{1}$H and $^{15}$N nuclei in red and blue triangles, respectively. The data were fitted assuming two identical binding sites (solid lines) and averaged to estimate a dissociation constant ($K_D$) of ~8.1 µM.

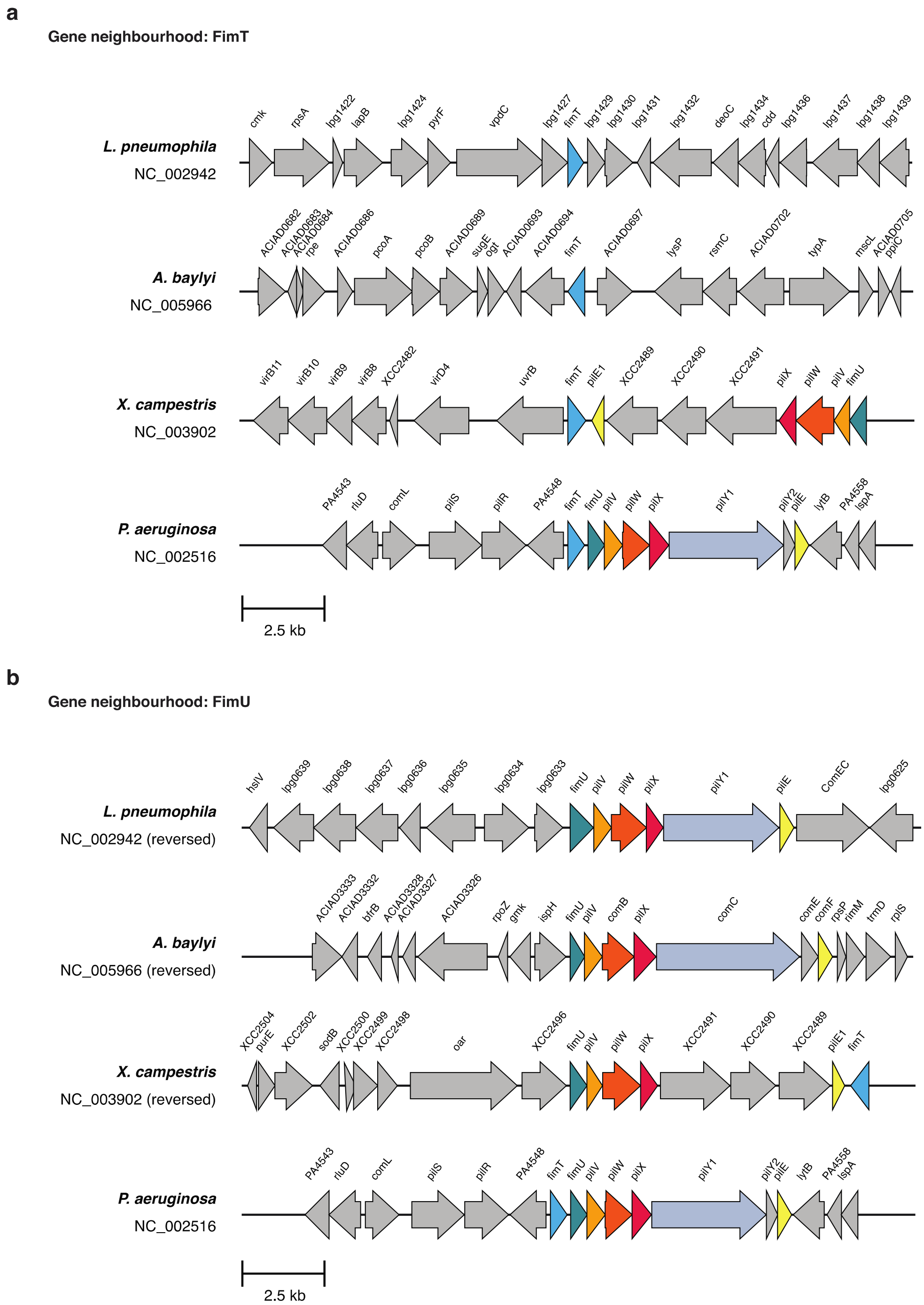


**Supplementary Figure 6: Gene neighbourhoods of *fimT* and *fimU***

Genomic regions around *fimT* (**a**) and *fimU* (**b**) in *L. pneumophila*, *A. baylyi*, *X. campestris* and *P. aeruginosa*. Each gene is labelled with its name or locus tag (if unannotated). Genes coding for T4P homologues are colour-coded identically across the different bacterial species. Among FimT and FimU homologues collected by BlastP using the four representative sequences, 25% of FimT sequences were located close to other minor pilin operon components, while 100% of FimU sequences were located in minor pilin operons. Source data are provided as a Source Data file.

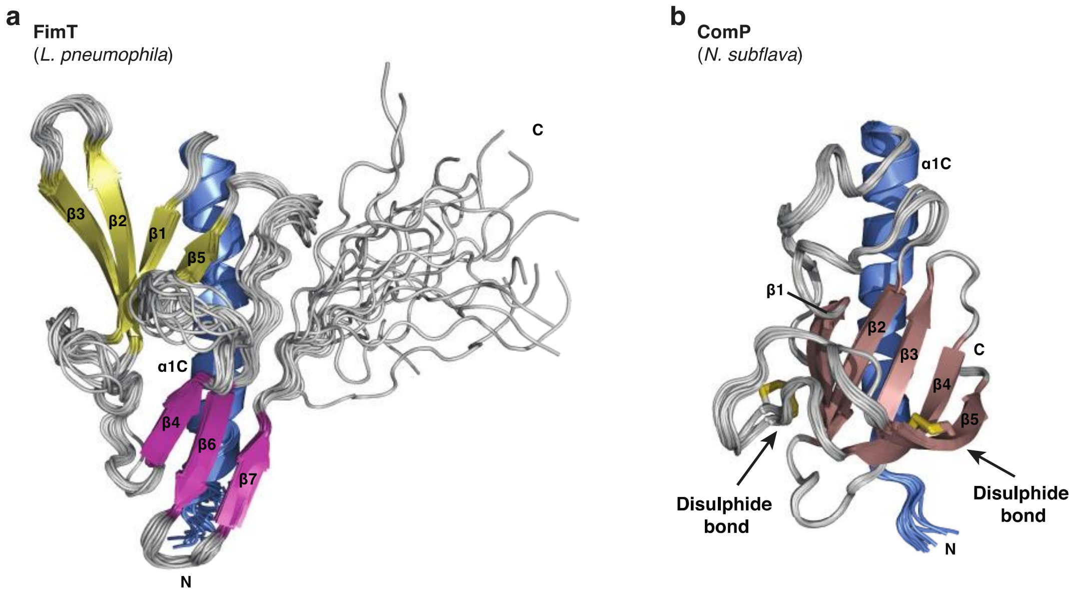


**Supplementary Figure 7: Comparison of the NMR structures of FimT and ComP**

**a**, **b**, Superimposed 20 lowest energy structures calculated by NMR spectroscopy of FimT from *L. pneumophila* (**a**) and ComP from *Neisseria subflava* (PDB ID: 2NBA[3]) (**b**). The DD-region defining disulphide bonds of ComP are shown in stick representation (sulphur atoms in yellow) and are indicated by arrows. Both structures are shown in ribbon representation with their N-and C-termini indicated.

**Supplementary Table 1 NMR and refinement statistics for $FimT_{Lp}$**

| | **$FimT_{Lp}$** |
|---|---|
| **NMR distance and dihedral constraints** | |
| Distance constraints | |
| Total NOE | 2311 |
| Intra-residue | 635 |
| Inter-residue | 1676 |
| Sequential ($\|i - j\| = 1$) | 522 |
| Medium-range ($\|i - j\| < 4$) | 344 |
| Long-range ($\|i - j\| > 5$) | 810 |
| Hydrogen bonds | - |
| Total dihedral angle restraints* | |
| Backbone | 666 |
| Other | 558 |
| **Structure statistics** | |
| Average Cyana target function | 0.21 ± 0.02 |
| Violations (mean and s.d.)** | |
| Distance constraints (Å) | 0 |
| Max. dihedral angle violation (º) | 123.98 ± 28.54 |
| Max. distance constraint violation (Å) | 0.57 ± 0.19 |
| Deviations from idealized geometry | |
| Bond lengths (Å) | 0.0035 ± 0.0012 |
| Bond angles (º) | 1.377 ± 0.459 |
| Average pairwise r.m.s. deviation*** (Å) | |
| Heavy | 1.13 ± 0.13 |
| Backbone | 0.56 ± 0.16 |

* Dihedral angle restraints were derived from Cα chemical shifts using TALOS+ as implemented in cyana 3.98

** Restraints violated in 6 or more structures

*** Pairwise r.m.s. deviation for structured regions (residues 32–62, 70–139) was calculated among 20 refined structures.

**Supplementary Table 2:** Strains used in this study

| Name | Relevant genotype/description | Source/Reference |
|---|---|---|
| *Escherichia coli* | | |
| BL21 (DE3) | *E. coli expression strain* | NEB (cat. no. C2527H/I) |
| Shuffle T7 | *E. coli expression strain* | NEB (cat. no. C3026J) |
| Stellar (HST08 strain) | *E. coli* cloning strain | Takara (cat. no. 636763/636766) |
| DH5α λpir | *E. coli cloning strain:* Encodes π protein for the replication of the *pir*-dependent origin of replication - *oriR*(*R6K)* | 4,5 |
| CR019 | *E. coli mobilising strain:* MT607 *E. coli* containing pRK600 plasmid [*oriR*(ColE1) *oriT*(RK2); CmR] | 6 |
| *Legionella pneumophila* | | |
| Lp02 WT | Philadelphia-1 *rpsL hsdR thyA*; SmR | 7 |
| Lp02 Δ*fimT* | Lp02 Δ*fimT (lpg1428)* | This study |
| Lp02 Δ*fimU* | Lp02 Δ*fimU (lpg0632)* | This study |
| Lp02 Δ*pilQ* | Lp02 Δ*pilQ (lpg0931)* | This study |
| Lp02 Δ*pilT* | Lp02 Δ*pilT (lpg2013)* | This study |
| Lp02 Δ*comEC* | Lp02 Δ*comEC (lpg0626)* | This study |

**Supplementary Table 3:** Plasmids used in this study

| Name | Relevant genotype/description | Source/Reference |
|---|---|---|
| pMMB207C | *Legionella* expression vector derived from RSF1010: IncQ lacI$^{q}$ P*tac oriT* Δ*mobA;* CmR | 8 |
| pMMB207C-*fimT$_{Lp}$* | *L. pneumophila* wild-type *fimT* | This study |
| pMMB207C-*fimT$_{Lp}$ R143Q* | pMMB207C-*fimT$_{Lp}$, with fimT R143Q mutation* | This study |
| pMMB207C-*fimT$_{Lp}$ R146Q* | pMMB207C-*fimT$_{Lp}$, with fimT R146Q mutation* | This study |
| pMMB207C-*fimT$_{Lp}$ R148Q* | pMMB207C-*fimT$_{Lp}$, with fimT R148Q mutation* | This study |
| pMMB207C-*fimT$_{Lp}$ R146Q, R148Q* | pMMB207C-*fimT$_{Lp}$*, with *fimT R146Q, R148Q mutations* | This study |
| pMMB207C-*fimT$_{Lp}$ R143Q, R146Q, R148Q* | pMMB207C-*fimT$_{Lp}$, with fimT R143Q, R146Q, R148Q mutations* | This study |
| pMMB207C-*fimT$_{Lp}$ S107Q* | pMMB207C-*fimT$_{Lp}$, with fimT S107Q mutation* | This study |
| pMMB207C-*fimT$_{Lp}$ S122Q* | pMMB207C-*fimT$_{Lp}$, with fimT S122Q mutation* | This study |
| pMMB207C-*fimT$_{Lp}$ G150Q* | pMMB207C-*fimT$_{Lp}$, with fimT G150Q mutation* | This study |
| pMMB207C-*fimT$_{Pa}$* | *Pseudomonas aeruginosa PAO1 fimT (PA4549)* | This study |
| pMMB207C-*fimT*$_{\text{chimera 1}}$ | pMMB207C-*fimT$_{Lp}$* residues 1-128, fused to *fimT$_{Pa}$* residues 125-161 | This study |
| pMMB207C-*fimT$_{Xc}$* | *Xanthomonas campestris ATCC 33913 fimT (XCC2486)* | This study |
| pMMB207C-*fimT*$_{\text{chimera 2}}$ | pMMB207C-*fimT$_{Lp}$* residues 1-128, fused to *fimT$_{Xc}$* residues 138-172 | This study |
| pSR47S | Suicide plasmid: *oriR*(*R6K) oriT*(RP4) *sacB*; KanR | 9 |
| pSR47S-*fimT* | *L. pneumophila fimT* gene with 1000 bp up- and downstream sequence (homology regions) | This study |
| pSR47S-*fimU* | *L. pneumophila fimU* gene with 1000 bp up- and downstream sequence (homology regions) | This study |
| pSR47S-*pilQ* | *L. pneumophila pilQ* gene with 1000 bp up- and downstream sequence (homology regions) | This study |
| pSR47S-*pilT* | *L. pneumophila pilT* gene with 1000 bp up- and downstream sequence (homology regions) | This study |
| pSR47S-*comEC* | *L. pneumophila comEC* gene with 1000 bp up- and downstream sequence (homology regions) | This study |
| pSR47S-Δ*fimT* | pSR47S-*fimT*, with *fimT* deletion | This study |
| pSR47S-Δ*fimU* | pSR47S-*fimU*, with *fimU* deletion (52 nt left intact at 5' end of gene) | This study |
| pSR47S-Δ*pilQ* | pSR47S-*pilQ*, with *pilQ* deletion | This study |
| pSR47S-Δ*pilT* | pSR47S-*pilT*, with *pilT* deletion | This study |
| pSR47S-Δ*comEC* | pSR47S-*comEC*, with *comEC* deletion | This study |
| pOPINS | *E. coli* expression vector: N-terminal $His_6$-SUMO tag, T7 promoter; KanR | 10 |
| pOPINS-*fimT$_{Lp}$* | *L. pneumophila wild-type fimT, residues 28-152* | This study |
| pOPINS-*fimT$_{Lp}$ K103Q* | pOPINS-*fimT$_{Lp}$*, *with fimT K103Q mutation* | This study |
| pOPINS-*fimT$_{Lp}$ R119Q* | pOPINS-*fimT$_{Lp}$*, *with fimT R119Q mutation* | This study |
| pOPINS-*fimT$_{Lp}$ R143Q* | pOPINS-*fimT$_{Lp}$*, *with fimT R143Q mutation* | This study |
| pOPINS-*fimT$_{Lp}$ R146Q* | pOPINS-*fimT$_{Lp}$*, *with fimT R146Q mutation* | This study |
| pOPINS-*fimT$_{Lp}$ R148Q* | pOPINS-*fimT$_{Lp}$*, *with fimT R148Q mutation* | This study |
| pOPINS-*fimT$_{Lp}$ R146Q, R148Q* | pOPINS-*fimT$_{Lp}$*, with *fimT R146Q, R148Q mutations* | This study |

| | | |
|---|---|---|
| pOPINS-*fimT*$_{Lp}$ *R143Q, R146Q, R148Q* | pOPINS-*fimT*$_{Lp}$, *with fimT R143Q, R146Q, R148Q mutations* | This study |
| pOPINS-*fimU*$_{Lp}$ | *L. pneumophila fimU, residues 28-167* | This study |
| pOPINS-*fimT*$_{Pa}$ | *Pseudomonas aeruginosa PAO1 fimT (PA4549),* residues 28-161 | This study |
| pOPINS-*fimU*$_{Pa}$ | *P. aeruginosa PAO1 fimU (PA4550),* residues 28-159 | This study |
| pOPINS-*fimT*$_{Xc}$ | *Xanthomonas campestris ATCC 33913 fimT (XCC2486),* residues 28-172 | This study |
| pOPINS-*fimU*$_{Xc}$ | *X. campestris ATCC 33913 fimU (XCC2495),* residues 28-163 | This study |
| pOPINB | *E. coli* expression vector: N-terminal $His_6$-tag, T7 promoter; KanR | 11 |
| pOPINB-*pilA1*$_{Lp}$ | *L. pneumophila pilA1 (lpg1914), residues 25-132* | This study |
| pOPINB-*pilA2*$_{Lp}$ | *L. pneumophila pilA2,* residues 25-131 | This study |
| pTRC99A | P*trc oriR*(pBR322); AmpR | 12 |
| pTRC99A-*lpg2953-2958::Kan* | *L. pneumophila* genomic region spanning *lpg2953-2958*. The *hipB* gene (*lpg2955*) is interrupted by kanamycin cassette, KanR | This study |

**Supplementary Table 4:** Oligonucleotides used in this study

| **Name** | **Sequence (5' to 3')** | **Construct** |
|---|---|---|
| *Cloning* | | |
| pMMB207_lin_F | aattcgagctcggtacccgg | pMMB207C |
| pMMB207_lin _R | ctgtttcctgtgtgaaattgttatccgc | |
| fimT$_{Lp}$_pMMB207C_F | tcacacaggaaacagatgcggcttcaattgatgaaaataacaggatt tac | pMMB207C-*fimT$_{Lp}$* |
| fimT$_{Lp}$_pMMB207C_R | taccgagctcgaattttaattaccccctaccctaaccctgcc | |
| fimT$_{Lp}$_R143Q_ pMMB207C_F | ccctaaccctgccaagctgatttaaagtaaccacaac | pMMB207C-*fimT$_{Lp}$* *R143Q* |
| fimT$_{Lp}$_R143Q_ pMMB207C_R | ggttactttaaatcagcttggcagggttagggtag | |
| fimT$_{Lp}$_R146Q_ pMMB207C_F | cttggccaggttagggtagggggtaattaaaattcg | pMMB207C-*fimT$_{Lp}$* *R146Q* |
| fimT$_{Lp}$_R146Q_ pMMB207C_R | taccctaacctggccaagcctatttaaagtaaccacaac | |
| fimT$_{Lp}$_R148Q_ pMMB207C_F | cagggttcaggtagggggtaattaaaattcgagctc | pMMB207C-*fimT$_{Lp}$* *R148Q* |
| fimT$_{Lp}$_R148Q_ pMMB207C_R | cccctacctgaaccctgccaagcctatttaaag | |
| fimT$_{Lp}$_R146QR148Q_ pMMB207C_F | cttggccaggttcaggtagggggtaattaaaattcg | pMMB207C-*fimT$_{Lp}$* *R146Q, R148Q* |
| fimT$_{Lp}$_R146QR148Q_ pMMB207C_R | cctacctgaacctggccaagcctatttaaagtaac | |
| fimT$_{Lp}$_ R143QR146QR148Q_ pMMB207C_F | gccaggttcaggtagggggtaattaaaattcgagctc | pMMB207C-*fimT$_{Lp}$* *R143Q, R146Q, R148Q* |
| fimT$_{Lp}$_ R143QR146QR148Q_ pMMB207C_R | ctacctgaacctggccaagctgatttaaagtaaccacaactttttcattg | |
| fimT$_{Lp}$_S107Q_ pMMB207C_F | gcgtagatcagaaccatagaattattatatccaatattccgaatcgtg | pMMB207C-*fimT$_{Lp}$* *S107Q* |
| fimT$_{Lp}$_S107Q_ pMMB207C_R | ctatggttctgatctacgcctttccaattaatattccaggaattag | |
| fimT$_{Lp}$_S122Q_ pMMB207C_F | gtgcgatgcagaatggtaaatttattttgaacaataagcgaaccaatg | pMMB207C-*fimT$_{Lp}$* *S122Q* |
| fimT$_{Lp}$_S122Q_ pMMB207C_R | ttaccattctgcatcgcacgattcggaatattggatataataattc | |
| fimT$_{Lp}$_G150Q_ pMMB207C_F | ttagggtacagggtaattaaaattcgagctcggtacccg | pMMB207C-*fimT$_{Lp}$* *G150Q* |
| fimT$_{Lp}$_G150Q_ pMMB207C_R | taattaccctgtaccctaaccctgccaagcctatttaaag | |
| fimT$_{Pa}$ _pMMB207C_F | tcacacaggaaacagatggtcgaaaggtcgcagagagc | pMMB207C-*fimT$_{Pa}$* |
| fimT$_{Pa}$ _pMMB207C_R | taccgagctcgaatttcatccggaagtgctgcatagctc | |
| fimT$_{Pa}$_125_pMMB207C_F | ggtaaatttattttgtgcggaaggcataccgttgc | pMMB207C-*fimT*$_{chimera\ 1}$ |
| fimT$_{Lp}$_138_pMMB207C_R | caaaataaatttaccattactcatcgcacgattcg | |
| *fimT$_{Xc}$* _pMMB207C_F | tcacacaggaaacagatgcagacaggacctcagtcacc | pMMB207C-*fimT$_{Xc}$* |
| *fimT$_{Xc}$* _pMMB207C_R | taccgagctcgaattttatgtctgcgcaggtgcc | |
| *fimT$_{Xc}$*_138_pMMB207C_F | ggtaaatttattttgtgcatccagtcagagcgagtgg | pMMB207C-*fimT*$_{chimera\ 2}$ |
| fimT$_{Lp}$_138_pMMB207C_R | caaaataaatttaccattactcatcgcacgattcg | |
| pSR47S_lin_F | ggatcccccgggctgcaggaattcg | pSR47S |
| pSR47S_lin_R | ccactagttctagagcggccgcc | |
| fimT_HR_pSR47S_F | ggccgctctagaactagtggtggcaaatgggatttaggtctccctcaa tg | pSR47S-*fimT* |
| fimT_HR_pSR47S_R | cctgcagcccgggggatccataaatgcctcagacaagctgacctctc c | |

| | | |
|---|---|---|
| fimU_HR_pSR47S_F | ggccgctctagaactagtggccaacacatcactaccttgttgagcattgcc | pSR47S-*fimU* |
| fimU_HR_pSR47S_R | tcctgcagcccgggggatcccaatcactattgatgatttgccctttgttggtg | |
| pilQ_HR_pSR47S_F | ggccgctctagaactagtggttgaaaaaaagcaacatcaggcagc | pSR47S-*pilQ* |
| pilQ_HR_pSR47S_R | tcctgcagcccgggggatccatcgaaacatcaacctcggcataaag | |
| pilT_HR_pSR47S_F | ggccgctctagaactagtggtatcgtaatgagtgcgaatattttctttactaatgc | pSR47S-*pilT* |
| pilT_HR_pSR47S_R | tcctgcagcccgggggatccccgttacaataacacgtaattttaccaattatgc | |
| comEC_HR_pSR47S_F | ggccgctctagaactagtggggtttatccacaaacattatcactgccactg | pSR47S-*comEC* |
| comEC_HR_pSR47S_R | tcctgcagcccgggggatccactctgcttgaaaggtatcccagg | |
| ΔfimT_HR_pSR47S_F | tcttaaattataagcaatggttgttcataaagagg | pSR47S-Δ*fimT* |
| ΔfimT_HR_pSR47S_R | ccattgcttataatttaagacatctacaaaattttatgatgaagataagatgcg | |
| ΔfimU_HR_pSR47S_F | agcattatccctattgtttgatcgaacccac | pSR47S-Δ*fimU* |
| ΔfimU_HR_pSR47S_R | caaacaatagggataatgctaacaacacccggccaagcagtc | |
| ΔpilQ_HR_pSR47S_F | tcaagattggactaattttatctcattaataaagataaaaaacattaatttaatagc | pSR47S-Δ*pilQ* |
| ΔpilQ_HR_pSR47S_R | ttagtccaatcttgagcctcactcctgc | |
| ΔpilT_HR_pSR47S_F | atacacatgacttgtgaaaaagacccaaggtc | pSR47S-Δ*pilT* |
| ΔpilT_HR_pSR47S_R | acaagtcatgtgtatactctataattcccgcc | |
| ΔcomEC_HR_pSR47S_F | atggattggctgacccatgttatatctaagc | pSR47S-Δ*comEC* |
| ΔcomEC_HR_pSR47S_R | ggtcagccaatccatttcaaattaagttggactttcc | |
| pOPINS_lin_F | taaagctttctagaccatttaaacaccaccac | pOPINS |
| pOPINS_lin_R | accaccgatctgttcgcgat | |
| fimT$_{Lp}$_28_pOPINS_F | atcgcgaacagatcggtggtatacaaaataatgagagagaaacattagttaatagtataaaaacagccattc | pOPINS-*fimT*$_{Lp}$ |
| fimT$_{Lp}$_152_pOPINS_R | aaatggtctagaaagctttattaattaccccctaccctaaccctgcc | |
| fimT$_{Lp}$_K103Q_pOPINS_F | tggaatattaattggcagggcgtagattcaaaccatag | pOPINS-*fimT*$_{Lp}$ *K103Q* |
| fimT$_{Lp}$_K103Q_pOPINS_R | tacgccctgccaattaatattccaggaattagaactcc | |
| fimT$_{Lp}$_R119Q_pOPINS_F | ccaatattccgaatcaggcgatgagtaatggtaaatttattttg | pOPINS-*fimT*$_{Lp}$ *R119Q* |
| fimT$_{Lp}$_R119Q_pOPINS_R | catcgcctgattcggaatattggatataataattctatggtttgaatc | |
| fimT$_{Lp}$_R143Q_pOPINS_F | ggttactttaaatcagcttggcagggttagggtag | pOPINS-*fimT*$_{Lp}$ *R143Q* |
| fimT$_{Lp}$_R143Q_pOPINS_R | ccctaaccctgccaagctgatttaaagtaaccacaac | |
| fimT$_{Lp}$_R146Q_pOPINS_F | gcttggccaggttagggtagggggtaattaataaag | pOPINS-*fimT*$_{Lp}$ *R146Q* |
| fimT$_{Lp}$_R146Q_pOPINS_R | cctaacctggccaagcctatttaaagtaaccacaac | |
| fimT$_{Lp}$_R148Q_pOPINS_F | cagggttcaggtagggggtaattaataaagctttctagac | pOPINS-*fimT*$_{Lp}$ *R148Q* |
| fimT$_{Lp}$_R148Q_pOPINS_R | cccctacctgaaccctgccaagcctatttaaagtaac | |
| fimT$_{Lp}$_R146QR148Q_pOPINS_F | cttggccaggttcaggtagggggtaattaataaagc | pOPINS-*fimT*$_{Lp}$ *R146Q, R148Q* |
| fimT$_{Lp}$_R146QR148Q_pOPINS_R | ccctacctgaacctggccaagcctatttaaagtaac | |
| fimT$_{Lp}$_R143QR146QR148Q_pOPINS_F | ggccaggttcaggtagggggtaattaataaagctttctag | pOPINS-*fimT*$_{Lp}$ *R143Q, R146Q, R148Q* |
| fimT$_{Lp}$_R143QR146QR148Q_pOPINS_R | tacctgaacctggccaagctgatttaaagtaaccac | |
| fimU$_{Lp}$_28_pOPINS_F | atcgcgaacagatcggtggtattttgaatagccgtttgacttcaaacattgac | pOPINS-*fimU*$_{Lp}$ |
| fimU$_{Lp}$_167_pOPINS_R | atggtctagaaagctttattaagggcagttcaaagctccattattcc | |
| fimT$_{Pa}$_28_pOPINS_F | atcgcgaacagatcggtggtctggacggcaatcgcgagc | pOPINS-*fimT*$_{Pa}$ |
| fimT$_{Pa}$_161_pOPINS_R | aaatggtctagaaagctttatcatccggaagtgctgcatagctc | |
| fimU$_{Pa}$_28_pOPINS_F | atcgcgaacagatcggtggtctgacagaacgcaacgaactgcag | pOPINS-*fimU*$_{Pa}$ |
| fimU$_{Pa}$_159_pOPINS_R | aaatggtctagaaagctttatcaatagcatgactggggcgc | |

| | | |
|---|---|---|
| fimT$_{Xc}$_28_pOPINS_F | atcgcgaacagatcggtggtatcgagcggcagcggttg | pOPINS-*fimT*$_{Xc}$ |
| fimT$_{Xc}$_172_pOPINS_R | aaatggtctagaaagctttattatgtctgcgcaggtgccgg | |
| fimU$_{Xc}$_28_pOPINS_F | atcgcgaacagatcggtggtattcggtcgaatcgcgctgttac | pOPINS-*fimU*$_{Xc}$ |
| fimU$_{Xc}$_163_pOPINS_R | aaatggtctagaaagctttatcattgacagttatcctttctacttctgactt gc | |
| pOPINB_lin_F | agcagcggtctggaagttctgtttcag | pOPINB |
| pOPINB_lin_R | atggtctagaaagcttta | |
| pilA1$_{Lp}$_25_pOPINB_F | aagttctgtttcagggcccgcaggactataccatcagagcac | pOPINB-*pilA1*$_{Lp}$ |
| pilA1$_{Lp}$_132_pOPINB_R | atggtctagaaagctttattaagggcggcagtagg | |
| pilA2$_{Lp}$_28_pOPINB_F | aagttctgtttcagggcccgcaagattacacaatacgagctcg | pOPINB-*pilA2*$_{Lp}$ |
| pilA2$_{Lp}$_131_pOPINB_R | atggtctagaaagctttattatggtctgcaactggcag | |
| pTRC99A_lin_F | gtgtctagagtcgacctgcaggcat | pTRC99A |
| pTRC99A_lin_R | gaacacaccagagatatctggcagaattc | |
| Lpg2953_F | atctctggtgtgttcggatagattatgcgagaggtctatttgaagattctct gactatg | pTRC99A-*lpg2953-2958::Kan* Amplification of transforming DNA |
| Lpg2958_R | gtcgactctagacacagacatggcctggaaacgttggtggg | |
| KanR_lin_F | cattcaaatatgtatccgctcatga | pTRC99A-*lpg2953-2958::Kan* |
| KanR_lin_R | cggggtctgacgctcagt | |
| pTRC99A_lpg2953_F | atacatatttgaatgcacgaatttctattctttggcc | pTRC99A-*lpg2953-2958::Kan* |
| pTRC99A_lpg2958_R | gagcgtcagaccccggctttggcagtttttctcttca | |
| | | |
| *DNA-binding assays** | | |
| FAM-12mer | # gttcgcaacgaa | MST/TRIC |
| 12mer | gttcgcaacgaa | NMR titrations/ITC |
| 30mer | ttaaataggcttggcagggttagggtaggg | EMSA |

* The complementary strand for dsDNA probes is not shown.
\# Indicates the position of the fluorescein (FAM) label. Only one of the two strands is FAM-labelled.

**Supplementary Table 5:** Gene locus tags of *fimT* and *fimU* genes from previous and recently updated genomes

| | ***L. pneumophila*** **Philadelphia 1 (old)*** | ***L. pneumophila*** **Philadelphia 1 (new)** | ***X. campestris*** **ATCC 33913 (old)*** | ***X. campestris*** **ATCC 33913 (new)** | ***A. baylyi*** **ADP1 (old)*** | ***A. baylyi ADP1 (new)*** |
|---|---|---|---|---|---|---|
| **RefSeq** | NC_002942.5 | NC_002942 | NC_003902.1 | NC_003902 | NC_005966.1 | NC_005966 |
| **Release date** | 2014 | 2021 | 2014 | 2021 | 2015 | 2020 |
| *fimT* | lpg1428 | LPG_RS07155 | XCC2486 | XCC_RS12930 | ACIAD0695 | ACIAD_RS03200 |
| *fimU* | lpg0632 | LPG_RS03130 | XCC2495 | XCC_RS12975 | ACIAD3321 | ACIAD_RS15030 |

* In this study we have referred to the old locus tags throughout.

**Supplementary Table 6:** Gene locus tags of selected genes from this study from previous and recently updated genomes

| | ***L. pneumophila*** **Philadelphia 1 (old)*** | ***L. pneumophila*** **Philadelphia 1 (new)** |
|---|---|---|
| **RefSeq** | NC_002942.5 | NC_002942 |
| **Release date** | 2014 | 2021 |
| *pilQ* | lpg0931 | LPG_RS04620 |
| *pilT* | lpg2013 | LPG_RS10105 |
| *comEC* | lpg0626 | LPG_RS03100 |
| *pilA1* | lpg1914 | LPG_RS09600 |
| *pilA2* | lpg1915 | LPG_RS09605 |
| *hipB* | lpg2955 | LPG_RS14950 |
| *pilV* | lpg0631 | LPG_RS03125 |
| *pilW* | lpg0630 | LPG_RS03120 |
| *pilX* | lpg0629 | LPG_RS03115 |
| *pilY1* | lpg0628 | LPG_RS03110 |
| *pilE* | lpg0627 | LPG_RS03105 |

* In this study we have referred to the old locus tags throughout.

## References


1. Yanez, M. E., Korotkov, K. K., Abendroth, J. & Hol, W. G. J. Structure of the Minor Pseudopilin EpsH from the Type 2 Secretion System of *Vibrio cholerae*. *Journal of Molecular Biology* **377**, 91–103 (2008).

2. Landau, M. *et al.* ConSurf 2005: the projection of evolutionary conservation scores of residues on protein structures. *Nucleic Acids Res* **33**, W299–W302 (2005).

3. Berry, J.-L. *et al.* A Comparative Structure/Function Analysis of Two Type IV Pilin DNA Receptors Defines a Novel Mode of DNA Binding. *Structure* **24**, 926–934 (2016).

4. Zuckman, D. M., Hung, J. B. & Roy, C. R. Pore-forming activity is not sufficient for *Legionella pneumophila* phagosome trafficking and intracellular growth. *Molecular microbiology* **32**, 990–1001 (1999).

5. Kolter, R., M, I. & R, H. D. Trans-Complementation-Dependent Replication of a Low Molecular Weight Origin Fragment from Plasmid R6K. *Cell* **15**, 1199–1208 (1978).

6. Finan, T. M., Kunkel, B., Vos, G. F. D. & Signer, E. R. Second Symbiotic Megaplasmid in *Rhizobium meliloti* Carrying Exopolysaccharide and Thiamine Synthesis Genes. *Journal of bacteriology* **167**, 66–72 (1986).

7. Berger, K. H. & Isberg, R. R. Two distinct defects in intracellular growth complemented by a single genetic locus in *Legionella pneumophila*. *Molecular microbiology* **7**, 7–19 (1993).

8. Chen, J. *et al. Legionella* Effectors That Promote Nonlytic Release from Protozoa. *Science* **303**, 1358–1361 (2004).

9. Merriam, J. J., Mathur, R., Maxfield-Boumil, R. & Isberg, R. R. Analysis of the *Legionella pneumophila* fliI Gene: Intracellular Growth of a Defined Mutant Defective for Flagellum Biosyntheis. *Infection and immunity* **65**, 2497–2501 (1997).

10. Assenberg, R. *et al.* Expression, purification and crystallization of a lyssavirus matrix (M) protein. *Acta Crystallogr Sect F Struct Biology Cryst Commun* **64**, 258–262 (2008).

11. Berrow, N. S. *et al.* A versatile ligation-independent cloning method suitable for high-throughput expression screening applications. *Nucleic Acids Research* **35**, e45–e45 (2007).

12. Amann, E., Ochs, B. & Abel, K.-J. Tightly regulated tac promoter vectors useful for the expression of unfused and fused proteins in Escherichia coli. *Gene* **69**, 301–315 (1988).
