# Supplementary material for: The molecular basis of FimT-mediated DNA uptake during bacterial natural transformation
Source: Nat Commun. 2022 Mar 4;13:1065. doi: 10.1038/s41467-022-28690-1 (PMC8897410; doi:10.1038/s41467-022-28690-1)
Supplement: Supplementary file 2 — Reporting Summary [file 41467_2022_28690_MOESM2_ESM.pdf]

nature portfolio

Corresponding author(s): Manuela K. Hospenthal

Last updated by author(s): 04.02.2022

Reporting Summary

Nature Portfolio wishes to improve the reproducibility of the work that we publish. This form provides structure for consistency and transparency in reporting. For further information on Nature Portfolio policies, see our Editorial Policies and the Editorial Policy Checklist.

Statistics

For all statistical analyses, confirm that the following items are present in the figure legend, table legend, main text, or Methods section.

n/a	Confirmed	
☐	☒	The exact sample size (*n*) for each experimental group/condition, given as a discrete number and unit of measurement
☐	☒	A statement on whether measurements were taken from distinct samples or whether the same sample was measured repeatedly
☐	☒	The statistical test(s) used AND whether they are one- or two-sided<br>*Only common tests should be described solely by name; describe more complex techniques in the Methods section.*
☒	☐	A description of all covariates tested
☒	☐	A description of any assumptions or corrections, such as tests of normality and adjustment for multiple comparisons
☐	☒	A full description of the statistical parameters including central tendency (e.g. means) or other basic estimates (e.g. regression coefficient) AND variation (e.g. standard deviation) or associated estimates of uncertainty (e.g. confidence intervals)
☐	☒	For null hypothesis testing, the test statistic (e.g. *F*, *t*, *r*) with confidence intervals, effect sizes, degrees of freedom and *P* value noted<br>*Give P values as exact values whenever suitable.*
☒	☐	For Bayesian analysis, information on the choice of priors and Markov chain Monte Carlo settings
☒	☐	For hierarchical and complex designs, identification of the appropriate level for tests and full reporting of outcomes
☒	☐	Estimates of effect sizes (e.g. Cohen's *d*, Pearson's *r*), indicating how they were calculated

Our web collection on statistics for biologists contains articles on many of the points above.

Software and code

Policy information about availability of computer code

Data collection	Topspin (versions 3.6.2 and 4.0.7)
Data analysis	CARA v1.9.1.8a2, CcpNmr v2.5.1, CYANA v3.98.13, Amber20, PyMOL v2.4.1, fitKD v1.3.0, GraphPad Prism v9, Origin v7.0383, Affinity Analysis (MST) v2.0.2, Blast v2.11.0, Custom NCBI script (https://github.com/francesca-short/NCBI_scripts), HMMER v3.3.1, MUSCLE v3.8.31, JalView v2.11.1.7, TrimAL v1.4.1, IQtree v1.6.12, iTol v5, Clinker v0.0.20, WebLogo v2.8.2

For manuscripts utilizing custom algorithms or software that are central to the research but not yet described in published literature, software must be made available to editors and reviewers. We strongly encourage code deposition in a community repository (e.g. GitHub). See the Nature Portfolio guidelines for submitting code & software for further information.

Data

Policy information about availability of data

All manuscripts must include a data availability statement. This statement should provide the following information, where applicable:

- Accession codes, unique identifiers, or web links for publicly available datasets
- A description of any restrictions on data availability
- For clinical datasets or third party data, please ensure that the statement adheres to our policy

The data that support the findings of this study are available from the corresponding author upon reasonable request. NMR spectra and corresponding model coordinates have been deposited in the BioMag Resonance Data Bank (BMRB: 34704) and Protein Data Bank (PDB ID: 7QYI), respectively. Source data are provided with this paper.

Field-specific reporting

Please select the one below that is the best fit for your research. If you are not sure, read the appropriate sections before making your selection.

[x] Life sciences [ ] Behavioural & social sciences [ ] Ecological, evolutionary & environmental sciences

For a reference copy of the document with all sections, see nature.com/documents/nr-reporting-summary-flat.pdf

Life sciences study design

All studies must disclose on these points even when the disclosure is negative.

Sample size	The sample size was stated in the figure legends for each experiment. We used sample sizes that are standard in our field. An example of another study having used the same approach is Vesel & Blokesch, Journal of Bacteriology, 2021.
Data exclusions	No data were excluded from our analyses.
Replication	Proper biological replicates were used in all experiments to ensure reproducibility. In addition, biological replicates were performed on separate days and always produced very similar results.
Randomization	We did not randomize the experimental groups. The knockout strains compared in our experiments were derived from the same parental strain.
Blinding	We did not perform blinding in all experiments. This is not standard for the types of experiments presented in our study.

Reporting for specific materials, systems and methods

We require information from authors about some types of materials, experimental systems and methods used in many studies. Here, indicate whether each material, system or method listed is relevant to your study. If you are not sure if a list item applies to your research, read the appropriate section before selecting a response.

Materials & experimental systems

n/a	Involved in the study	
[x]	[ ]	Antibodies
[x]	[ ]	Eukaryotic cell lines
[x]	[ ]	Palaeontology and archaeology
[x]	[ ]	Animals and other organisms
[x]	[ ]	Human research participants
[x]	[ ]	Clinical data
[x]	[ ]	Dual use research of concern

Methods

n/a	Involved in the study	
[x]	[ ]	ChIP-seq
[x]	[ ]	Flow cytometry
[x]	[ ]	MRI-based neuroimaging
